# The synergistic effect of obesity and dyslipidemia on hypertension: results from the STEPS survey

**DOI:** 10.1186/s13098-024-01315-x

**Published:** 2024-04-03

**Authors:** Parisa Mohseni, Davood Khalili, Shirin Djalalinia, Hamideh Mohseni, Farshad Farzadfar, Arman Shafiee, Neda Izadi

**Affiliations:** 1 Department of Epidemiology, School of Public Health and Safety, Shahid Beheshti University of Medical Sciences, Tehran, Islamic Republic of Iran; 2 Prevention of Metabolic Disorders Research Center, Research Institute for Endocrine Sciences, Shahid Beheshti University of Medical Sciences, Tehran, Islamic Republic of Iran; 3 Non-Communicable Diseases Research Center, Endocrinology and Metabolism Population Sciences Institute, Tehran University of Medical Sciences, Tehran, Islamic Republic of Iran; 4 Islamic Azad University of Larestan, Lar, Islamic Republic of Iran; 5 Endocrinology and Metabolism Research Center, Endocrinology and Metabolism Clinical Sciences Institute, Tehran University of Medical Sciences, Tehran, Islamic Republic of Iran; 6 School of Medicine, Alborz University of Medical Sciences, Alborz, Islamic Republic of Iran; 7 Research Center for Social Determinants of Health, Research Institute for Endocrine Sciences, Shahid Beheshti University of Medical Sciences, Tehran, Islamic Republic of Iran

**Keywords:** Obesity, Dyslipidemia, Hypertension, STEPS survey, Synergy index

## Abstract

**Background:**

Obesity and dyslipidemia are important risk factors for hypertension (HTN). When these two conditions coexist, they may interact in a synergistic manner and increase the risk of developing HTN and its associated complications. The aim of this study was to investigate the synergistic effect of general and central obesity with dyslipidemia on the risk of HTN.

**Method:**

Data from 40,387 individuals aged 25 to 64 years were obtained from a repeated cross-sectional study examining risk factors for non-communicable diseases (STEPS) in 2007, 2011 and 2016. Body mass index (BMI) was calculated as a measure of general obesity and waist circumference (WC) as a measure of central obesity. Dyslipidemia was defined as the presence of at least one of the lipid abnormalities. Hypertension was defined as systolic blood pressure ≥ 140 mmHg or diastolic blood pressure ≥ 90 mmHg or current use of antihypertensive medication. To analyze the synergistic effect between obesity and dyslipidemia and HTN, the relative excess risk due to interaction (RERI), attributable proportion due to interaction (AP), and synergy index (SI) were calculated. A weighted logistic regression model was performed to estimate the odds ratios (ORs) for the risk of HTN.

**Results:**

The results showed an association between obesity, dyslipidemia and hypertension. The interaction between obesity and dyslipidemia significantly influences the risk of hypertension. In hypertensive patients, the presence of general obesity increased from 14.55% without dyslipidemia to 64.36% with dyslipidemia, while central obesity increased from 13.27 to 58.88%. This interaction is quantified by RERI and AP values of 0.15 and 0.06 for general obesity and 0.24 and 0.09 for central obesity, respectively. The corresponding SI of 1.11 and 1.16 indicate a synergistic effect. The OR also show that the risk of hypertension is increased in the presence of obesity and dyslipidemia.

**Conclusion:**

Obesity and dyslipidemia are risk factors for HTN. In addition, dyslipidemia with central obesity increases the risk of HTN and has a synergistic interaction effect on HTN. Therefore, the coexistence of obesity and lipid abnormalities has many clinical implications and should be appropriately monitored and evaluated in the management of HTN.

## Introduction

Hypertension (HTN) affects more than one billion people worldwide and is a serious condition that significantly increases the risk of heart, brain and kidney disease and is the leading cause of premature mortality worldwide [1, 2]. The prevalence of HTN ranges from 4 to 78% in different regions of the world [3] and one of the global targets for non-communicable diseases (NCDs) is to reduce the prevalence of HTN by 33% between 2010 and 2030 [4].

The prevalence of HTN in Iran among adults over 25 years of age is estimated to be 31% in men and 27% in women [5]. Therefore, identifying risk factors for HTN is very important for optimal treatment of patients and prevention of complications.

To date, many studies have shown that obesity, dyslipidemia, and fasting plasma glucose (FPG) are strongly associated with HTN [6]. Therefore, both general obesity and central obesity can be used to identify HTN [7]. Studies in different populations around the world have shown that the relationship between body mass index (BMI) and systolic and diastolic blood pressure (SBP/DBP) is almost linear [8]. Risk estimates from the Framingham Heart Study show that 78% of baseline (essential) blood pressure (BP) in men and 65% in women can be attributed to overweight [8, 9].

Dyslipidemia, an abnormal amount of lipids in the blood, is common in hypertensive patients, and elevated serum levels of total cholesterol (TC), triglyceride (TG) and low-density lipoprotein cholesterol (LDL-C) are all associated with an increased risk of developing HTN [10]. Therefore, hypertension is a multifactorial disease in which the contributing factors interact with each other [6].

In general, physicians focus more on the association between risk factors and treatment outcomes. Obesity and dyslipidemia are known risk factors for HTN. When these two conditions coexist, they can interact in a synergistic manner, increasing the risk of developing HTN and its associated complications. Understanding the complex interplay between obesity, dyslipidemia and hypertension is crucial for developing effective prevention and treatment strategies to address these interrelated health problems.

Despite the existence of a similar published study, it is crucial to emphasize that further research is needed to deepen our understanding of this interaction. While the earlier study may have laid a foundation, our study used a larger sample size at a national level to examine the interaction between general obesity and central obesity, rather than just looking at BMI. Central obesity, particularly excess fat around the abdomen, is associated with a higher risk of metabolic complications such as insulin resistance, dyslipidemia and cardiovascular disease [11]. BMI, on the other hand, does not differentiate between different fat distribution patterns. Studies have shown that waist circumference (WC) is a better predictor of cardiovascular risk and mortality than BMI alone [11–13]. By incorporating WC measurements, healthcare providers can better assess a person’s overall health risk associated with obesity.

## Method

### Participants

In this repeated cross-sectional study, data were obtained from the STEPwise approach to non-communicable diseases (NCD) risk factor surveillance (STEPS) in 2007, 2011 and 2016, which included the Iranian population aged ≥ 18 years. In these surveys, individuals were selected using multistage cluster sampling that is representative of the urban and rural population of all provinces of Iran. The basis of the survey design has already been described in other studies [14–16]. After obtaining the written consent of the subjects, the data were collected by trained personnel during home interviews. The information included demographic data, lifestyle and risk factors such as smoking, diet, physical activity, hypertension and history of diabetes. The laboratory measurements were carried out on people over the age of 25. In this study, people under the age of 25 and people for whom laboratory tests (lipid profile) were not available were excluded, and finally, data from 40,387 people aged 25 to 64 were analyzed.

### Measurements

In STEPS survey, anthropometric variables (height, weight and WC) were measured by participants’ barefoot but wearing light clothing. Height and weight were measured using a scale with an accuracy of 1 cm and a weight of 1 kg (Innofit, China). The waist circumference of the participants was measured at the level of the iliac and umbilical processes using a tape measure with precision of 1 cm. BMI was calculated by dividing the weight by the square meter of height [17]. Physical activity was determined using the Global Physical Activity Questionnaire (GPAQ) [18]. Systolic and diastolic blood pressure were measured three times at 10-minute intervals using the device from Beurer GmbH, Germany, and the mean values of the second and third measurements were used for analysis. Fasting blood samples were collected from participants after an overnight fast of at least 12 h, centrifuged immediately and transferred under cold chain conditions to the NCD Research Center, the coordinating center of this study in Tehran. Fasting plasma glucose, TC, high-density lipoprotein cholesterol (HDL-C) and TG were measured using an autoanalyzer (Cobas C311 Hitachi High–Technologies Corporation, Japan) [17]. LDL-C was calculated using the Chen formula [19].

Physical activity was measured by 24 h physical activity and the GPAQ questionnaire. After calculating METS (metabolic equivalents) to represent the intensity of physical activity, individuals were categorized into three groups of low, moderate, and high physical activity. Smoking included people who currently smoked cigarettes daily. Diabetes was defined as FPG ≥ 126 mg/dL or use of blood glucose-lowering medication, or if they had previously been told by a healthcare professional that they had diabetes. The consumption of fruit, vegetables and dairy products expressed in units of consumption per day.

Physical activity level was categorized as low (≤ 600 MET min/week), moderate (600–3000 MET min/week) and high (≥ 3000 MET min/week). BMI was categorized into 4 levels: less than 18.5 kg/m^2^ as underweight, 18.5 to 24.9 kg/m^2^ as normal, between 25 and 29.9 kg/m^2^ as overweight, and over 30 kg/m^2^ as obese. General obesity was defined as a BMI of more than 30 kg/m^2^ and central obesity as WC greater than 88 cm in women and 102 cm in men [14]. Dyslipidemia was defined as hypercholesterolemia (TC ≥ 200 mg/dL), and/or hypertriglyceridemia (TG ≥ 150 mg/dL), and/or hyper-LDL (LDL ≥ 130 mg/dL) and/or hypo-HDL (HDL < 40 mg/dL in men and < 50 mg/dL in women) based on the National Cholesterol Education Program Adult Treatment Panel III (NCEP ATP III) classification of lipid profile [20]. Hypertension was defined as systolic blood pressure (SBP) ≥ 140 mmHg or diastolic blood pressure (DBP) ≥ 90 mmHg or current use of antihypertensive medication. Metabolic syndrome was defined as the presence of ≥ 3 of the following criteria based on the American Heart Association/National Heart definition:

(1) Waist circumference ≥ 102 cm in men and WC ≥ 88 cm in women (2) SBP ≥ 130 mmHg or DBP ≥ 85 mmHg or treatment with anti-hypertensive medication (3) TG ≥ 150 mg/dL or on treatment for high TG (4) HDL < 40 mg/dL in men and < 50 mg/dL in women or on treatment for low HDL (5) FPG ≥ 100 mg/dL or known diabetes [21].

### Statistical analysis

Analyzes were calculated after weighting to assimilate the sample studied due to differences in provincial populations. For this purpose, the 2006, 2011 and 2016 censuses were extracted from the National Statistics Portal (Iran) and weighting was applied by gender, age, region of residence, for all provinces and the sample (STEPS data). Finally, the weight of each province was determined by dividing its population by the sample population. Continuous variables were reported with the weighted mean (standard deviation (SD)) and categorical variables with the number (weighted percentage). Baseline characteristics were compared using ttest and chisquare. To analyze the synergism between obesity and dyslipidemia, and HTN the following indices were calculated: (1) Relative excess risk due to interaction (RERI); (2) Attributable proportion due to interaction (AP); (3) Synergy index (SI). Weighted logistic regression model was performed to estimate the crude and adjusted odds ratios (ORs) for the risk of HTN. The significant variables from the univariable model were included in the multivariable analysis. All data were calculated with a significance level of less than 0.05 and a confidence interval of 95% using STATA software (version 18).

This study was conducted according to the guidelines laid down in the Declaration of Helsinki and all procedures involving research study participants were approved by the Ethics Committee of Shahid Beheshti University of Medical Sciences (IR.SBMU.PHNS.REC.1399.059). Written informed consent was obtained from all subjects.

## Results

A total of 40,387 adults participated in this study, with 18,800 participants in 2007, 4,830 in 2011, and 16,757 in 2016. Among them, 18,444 (45.7%) were male and 21,943 (54.3%) were female. Out of the total sample, 15,704 (30.80%) individuals were from rural regions, and 24,683 (69.20%) were from urban regions. Data from the STEPS study (2007–2016) show that general obesity and central obesity were prevalent in 9,656 (23.91%) and 16,182 (40.07%) of the cohort, respectively. Dyslipidemia was found in 31,258 (77.41%) participants. The mean age was 41.56 years, with a higher proportion of obesity and dyslipidemia found in women than in men. These conditions were more common in urban areas than in rural areas. Those with general obesity had higher mean BMI, WC, BP and lipid levels, suggesting an association between obesity and dyslipidemia (Table 1).

The results showed that the mean age of hypertensive patients was 44.45 years, compared with 40.69 years in the normotensive group. The prevalence of hypertension was higher in men (45.85%) than in women (41.15%). The frequency of urban residents with hypertension was 70.37%, compared with 29.63% in rural areas. Those with hypertension had higher mean values for BMI (28.97 kg/m²), WC (95.99 cm), TC (188.80 mg/dL), TG (168.72 mg/dL), LDL-C (115.30 mg/dL), HDL-C (41.94 mg/dL) and FPG (103.73 mg/dL). Patients with diabetes were more common in the hypertensive group (18.82%). The prevalence of metabolic syndrome in the hypertensive and normotensive groups was 65.80% and 24.06% respectively. The consumption of fruit and vegetables was slightly lower in the participants with hypertension, with an average of 1.47 and 1.62 portions per day respectively (Table 2).

**Fig. 1:**
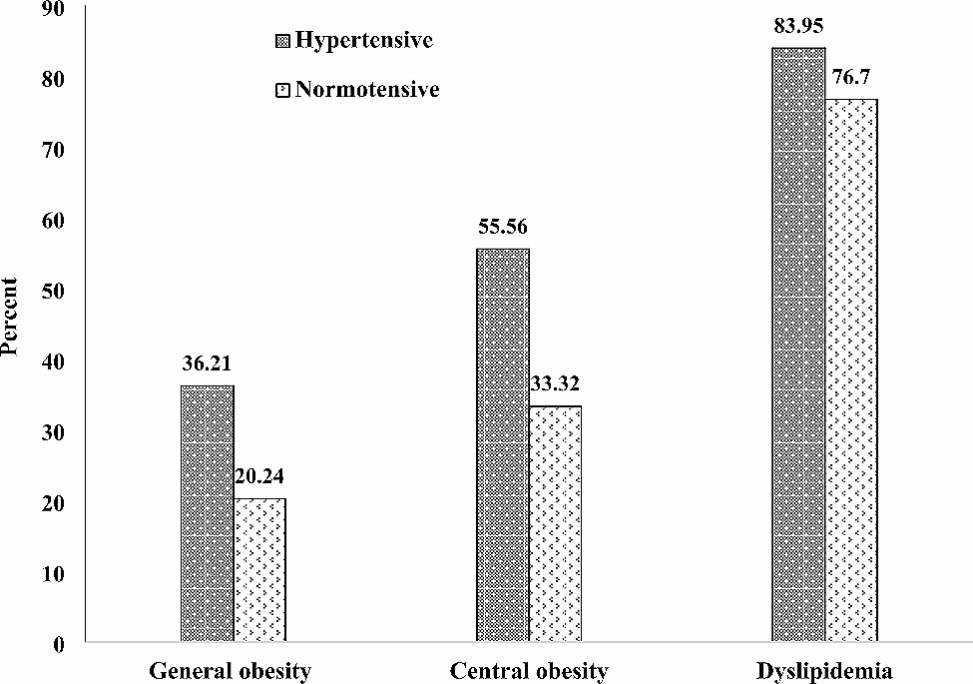
Frequency of obesity and dyslipidemia by hypertension in participants of the STEPS study from (2007–2016)

In the STEPS study from 2007 to 2016, the prevalence of general obesity in hypertensives was 36.21% compared to 20.24% in normotensive individuals. Central obesity was observed in 55.56% of participants with hypertension compared to 33.32% in their normotensive counterparts. Dyslipidemia was significantly higher in the hypertensive group with a prevalence of 83.95% compared to 76.7% in the normotensive group. These results show a clear association between obesity, dyslipidemia and hypertension in the study participants (Fig. 1).

The interaction between obesity and dyslipidemia on the risk of hypertension was examined in Table 3. In general obesity without dyslipidemia, the prevalence was 14.55% in the hypertensive group and 85.45% in the normotensive group. If both general obesity and dyslipidemia were present, the prevalence in the hypertensive group increased to 64.36%. The RERI was 0.15, the AP was 0.06 and the SI was 1.11. The prevalence of central obesity was 13.27% without dyslipidemia and 58.88% with dyslipidemia among hypertensives. The RERI for central obesity was 0.24, the AP was 0.09 and the SI was 1.16, indicating a positive interaction between central obesity and dyslipidemia on hypertension risk (Table 3).

**Fig. 2:**
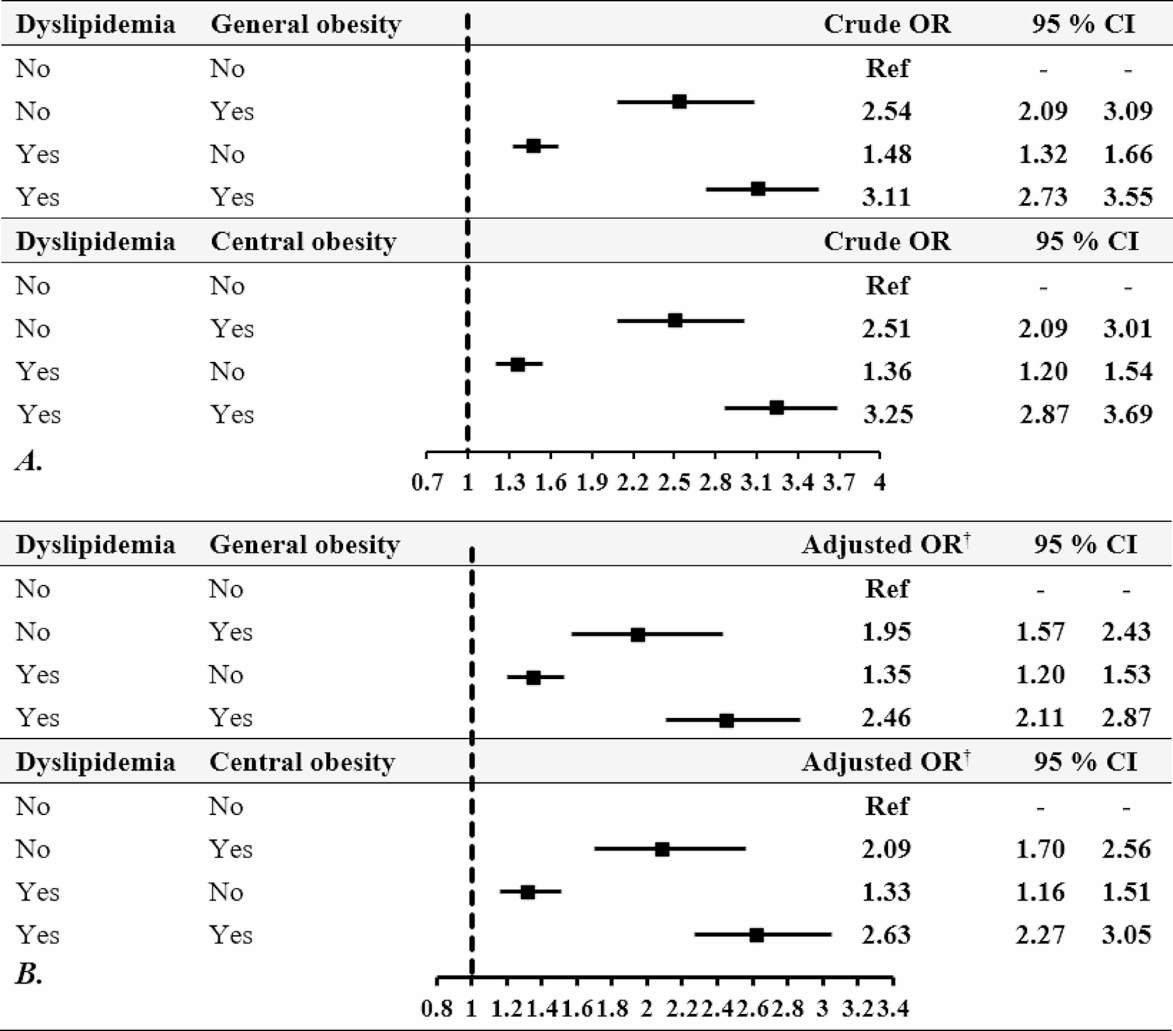
The interaction between obesity and dyslipidemia on the risk of hypertension using univariable (**A**) and multivariable (**B**) weighted logistic regression ^**†**^Adjusted for age, sex, physical activity, history of diabetes, and cooking oil type, OR = Odds Ratio; CI = Confidence Interval

Figure 2 shows the odds ratios for the risk of hypertension associated with obesity and dyslipidemia. For general obesity without dyslipidemia, the crude OR was 2.54 (95% CI: 2.09–3.09) and with dyslipidemia 3.11 (95% CI: 2.73–3.55). The adjusted OR considering multiple variables was 1.95 (95% CI: 1.57–2.43) without dyslipidemia and 2.46 (95% CI: 2.11–2.87) with dyslipidemia. For central obesity, the crude OR was 2.51 (95% CI: 2.09–3.01) without dyslipidemia and 3.25 (95% CI: 2.87–3.69) with dyslipidemia, while the adjusted OR was 2.09 (95% CI: 1.70–2.56) without dyslipidemia and 2.63 (95% CI: 2.27–3.05) with dyslipidemia, indicating a stronger association of both types of obesity with hypertension when combined with dyslipidemia.

**Table 1:** Descriptive characteristics of participants in the STEPS study from 2007 to 2016, by obesity and dyslipidemia

Variables	Total (*n* = 40,387)	General obesity (BMI)		Central obesity (WC)		Dyslipidemia	
		**Yes** **(** *n* ** = 9,656)**	**No** **(** *n* ** = 30,051)**	**Yes** **(** *n* ** = 16,182)**	**Yes** **(** *n* ** = 23,564)**	**Yes** **(** *n* ** = 32,258)**	**No** **(** *n* ** = 8,129)**
Age (year)	41.56 (11.44)	44.45 (10.84)	40.69 (11.46)	44.98 (11.08)	39.53 (11.15)	41.94 (11.41)	40.15 (11.42)
Sex^†^							
Male	18,444 (46.90)	2,788 (31.39)	15,436 (52.12)	3,402 (23.60)	14,846 (61.85)	13,835 (44.22)	4,609 (56.65)
Female	21,943 (53.10)	6,868 (68.61)	14,615 (47.88)	12,780 (76.40)	8,718 (38.15)	18,423 (55.78)	3,520 (43.35)
Residence^†^							
Urban	24,683 (69.20)	6,608 (74.67)	17,670 (67.70)	10,634 (72.12)	13,674 (67.66)	20,140 (70.35)	4,543 (65.02)
Rural	15,704 (30.80)	3,048 (25.33)	12,381 (32.30)	5,548 (27.88)	9,890 (32.34)	12,118 (29.65)	3,586 (34.98)
Physical activity^†^							
Low	16,637 (42.12)	4,445 (44.05)	11,881 (41.07)	7,498 (44.60)	8,851 (40.04)	13,574 (42.99)	3,063 (38.93)
Moderate	9,508 (24.77 )	2,307 (24.45)	7,149 (25.05)	4,101 (26.57)	5,357 (23.85)	7,719 (25.01)	1,789 (23.91)
High	13,901 (33.11)	2,893 (31.50)	10,952 (33.88)	4,567 (28.82)	9,285 (36.11)	10,706 (32.00)	3,195 (37.16)
Smoking (yes)^†^	4,660 (11.63)	536 (6.10)	4,102 (13.56)	774 (5.39)	3,866 (15.73)	3,601 (11.51)	1,059 (12.06)
BMI (kg/m^2^)	26.79 (5.26)	33.83 (4.06)	24.59 (3.29)	30.90 (4.60)	24.25 (3.85)	27.34 (5.24)	24.82 (4.84)
WC (cm)	90.27 (13.59)	103.51 (10.76)	86.12 (11.57)	101.76 (9.95)	83.14 (10.26)	91.56 (13.44)	85.59 (13.11)
SBP (mmHg)	123.93 (18.21)	129.96 (19.22)	122.12 (17.48)	128.89 (19.44)	120.95 (16.71)	124.74 (18.57)	120.97 (16.50)
DBP (mmHg)	78.70 (11.84)	82.44 (11.94)	77.61 (11.56)	81.68 (11.96)	76.94 (11.39)	79.28 (11.99)	76.63 (11.06)
Hypertension (yes)^†^	10,199 (22.34)	3,530 (34.13)	6,632 (18.80)	5,629 (32.55)	4,540 (16.17)	8,690 (23.94)	1,509 (16.54)
TC (mg/dL)	175.90 (41.80)	172.35 (40.63)	187.35 (43.37)	185.95 (44.17)	169.70 (38.97)	180.30 (44.58)	159.91 (23.38)
TG (mg/dL)	146.51 (86.18)	169.91 (92.62)	139.36 (82.88)	162.96 (92.99)	136.51 (80.20)	162.04 (90.34)	90.12 (26.60)
LDL-C(mg/dL)	105.48 (33.99)	114.40 (34.89)	102.74 (33.24)	113.23 (35.51)	100.73 (32.13)	110.50 (35.19)	87.27 (20.71)
HDL-C (mg/dL)	42.44 (11.37)	41.41 (11.46)	42.74 (11.29)	42.08 (11.46)	42.62 (11.27)	39.55 (10.03)	52.94 (9.67)
FPG (mg/dL)	95.42 (31.88)	101.47 (35.38)	93.60 (30.51)	101.17 (36.32)	91.98 (28.34)	96.92 (33.69)	90.00 (23.37)
Diabetes (yes)	4,788 (10.85)	1,780 (17.63)	2,948 (8.80)	2,939 (17.46)	1,803 (6.87)	4,239 (12.26)	549 (5.70)
Cooking oil type^†^							
Liquid	19,191 (53.80)	5,049 (56.59)	13,872 (52.75)	8,256 (55.30)	10,687 (52.66)	15,488 (54.22)	3,703 (52.29)
Solid	20,288 (44.76)	4,447 (41.73)	15,709 (45.88)	7,662 (43.18)	12,507 (45.95)	16,035 (44.36)	4,253 (46.22)
Other	596 (1.44)	149 (1.68)	441 (1.36)	246 (1.51)	344 (1.39)	486 (1.42)	110 (1.49)
Fruits consumption(average per day)	1.89 (1.35)	1.92 (1.30)	1.88 (1.36)	1.88 (1.30)	1.89 (1.38)	1.90 (1.36)	1.83 (1.29)
Vegetables consumption (average per day)	1.60 (1.23)	1.64 (1.20)	1.60 (1.24)	1.62 (1.21)	1.60 (1.25)	1.62 (1.23)	1.56 (1.21)

Data are presented as Mean (Standard deviation), ^†^Frequency (% weighted), BMI: Body Mass Index, WC: Waist Circumference, SBP: Systolic Blood Pressure, DBP: Diastolic Blood Pressure, TC: Total Cholesterol, TG: Triglyceride, LDL-C: Low-Density Lipoprotein Cholesterol, HDL-C: High-Density Lipoprotein Cholesterol, FPG: Fasting Plasma Glucose

**Table 2:** Descriptive characteristics of participants in the STEPS study from 2007 to 2016, by hypertension

Variables		Hypertensive (*n* = 10,199)	Normotensive (*n* = 29,786)	P-value^*^
Age (year)		44.45 (10.84)	40.69 (11.46)	< 0.001
Sex^†^				
Male		4,530 (45.85)	13,722 (47.24)	< 0.001^**^
Female	5,669 (54.15)		16,064 (52.76)	
Residence^†^				
Urban		6,246 (70.37)	18,193 (69.04)	< 0.001^**^
Rural	3,953 (29.63)		11,593 (30.96)	
Physical activity^†^				
Low		4,170 (40.36)	12,379 (42.54)	< 0.001^**^
Moderate	2,515 (25.84)		6,958 (24.48)	
High	3,499 (33.80)		10,362 (32.97)	
Smoking (yes)^†^		1,002 (10.47)	3,638 (12.07)	< 0.001^**^
BMI (kg/m^2^)		28.87 (5.39)	26.19 (5.06)	< 0.001
WC (cm)		95.99 (13.11)	88.62 (13.28)	< 0.001
TC (mg/dL)		188.80 (44.80)	172.20 (40.12)	< 0.001
TG (mg/dL)		168.72 (96.15)	140.25 (82.08)	< 0.001
LDL-C (mg/dL)		115.30 (36.23)	102.64 (32.78)	< 0.001
HDL-C (mg/dL)		41.94 (11.22)	42.60 (11.41)	< 0.001
FPG (mg/dL)		103.73 (41.51)	93.02 (28.04)	< 0.001
Diabetes (yes)^†^		1,972 (18.82)	2,781 (8.60)	< 0.001^**^
MetS (yes)^†^		6,719 (65.80)	7,687 (24.06)	< 0.001^**^
Cooking oil type^†^				
Liquid		4,699 (51.80)	14,381 (54.27)	< 0.001^**^
Solid	5,343 (46.65)		14,908 (44.33)	
Other	147 (1.55)		445 (1.40)	
Fruits consumption (average per day)		1.84 (1.36)	1.90 (1.34)	0.001
Vegetables consumption (average per day)		1.62 (1.25)	1.60 (1.22)	< 0.001

Data were presented as Mean (Standard deviation), ^†^Frequency (% weighted), BMI: Body Mass Index, WC: Waist Circumference, TC: Total Cholesterol, TG: Triglyceride, LDL-C: Low-Density Lipoprotein Cholesterol, HDL-C: High-Density Lipoprotein Cholesterol, FPG: Fasting Plasma Glucose, MetS: Metabolic Syndrome, ^**^Based on T-test, ^**^Based on Chi-square

**Table 3:** Indicators of interaction between obesity, BMI and dyslipidemia on the risk of hypertension

Variables		Hypertensive (*n* = 10,169)	Normotensive (*n* = 29,464)
		**N (% weighted)**	**N (% weighted)**
**Dyslipidemia**	**General obesity**		
No	No	1,134 (14.55)	5,714 (85.45)
No	Yes	367 (30.23)	764 (69.77)
Yes	No	5,498 (20.20)	17,643 (79.80)
Yes	Yes	3,163 (34.66)	5,343 (65.34)
**Indicators of interaction** ^**†**^ **(95% CI)**	RERI	0.15 (-0.31-0.62)	
	AP	0.06 (-0.12-0.24)	
	SI	1.11 (0.79–1.55)	
**Dyslipidemia**	**Central obesity**		
No	No	934 (13.27)	5,135 (86.73)
No	Yes	565 (27.80)	1,348 (72.20)
Yes	No	3,606 (17.26)	13,832 (82.74)
Yes	Yes	5,064 (58.88)	9,176 (37.48)
**Indicators of interaction** ^**†**^ **(95% CI)**	RERI	0.24 (-0.18-0.67)	
	AP	0.09 (0.01–0.24)	
	SI	1.16 (1.007–1.36)	

^**†**^Adjusted for age and sex, RERI: Relative Excess Risk due to Interaction, AP: Attributable Proportion due to interaction, SI: Synergy Index, CI = Confidence Interval

## Discussion

This population-based cross-sectional study showed a synergistic effect between dyslipidemia and central obesity on blood pressure, such that obese individuals with dyslipidemia had a higher risk of developing hypertension. Similar studies have shown that dyslipidemia and obesity share common mechanisms for increasing blood pressure. In patients with dyslipidemia, the bioactivity of nitric oxide is impaired, resulting in reduced vasodilator capacity and increased blood pressure. In addition, atherosclerotic lesions caused by endothelial dysfunction are associated with increased arterial stiffness and decreased arterial compliance, leading to high blood pressure [22, 23]. Several mechanisms support the effects of weight gain on HTN. Mechanisms of this association include activation of the renin-angiotensin-aldosterone system by increasing sympathetic activity, insulin resistance, leptin resistance, increased procoagulant activity, and endothelial dysfunction, which may lead to HTN and cardiovascular disease [24].

The close association between obesity and blood pressure has long been recognized in both sexes and even in different racial/ethnic groups. However, the results of various studies show that the best anthropometric index to predict hypertension and other components of metabolic syndrome is inconclusive and controversial [25]. Some studies reported that BMI is the most sensitive physical measure for predicting HTN compared to other common obesity indicators [26].

In other studies, WC was found to be a better predictor in Greeks [27], Taiwanese [28], and some Japanese men [29]. Lee and Kim suggested that the combination of two or more indices may improve the predictive power of an index [30]. Therefore, the BMI and WC indices were examined in this study, and it was found that generally and centrally obese adults had higher BP than normal-weight individuals. This finding is consistent with the reports of previous studies [31, 32].

A study in Kenya showed that generally and centrally obese adults were twice as likely to have HTN and dyslipidemia and four times more likely to have multiple comorbidities than their normal weight and normal WC counterparts, regardless of gender [33]. In Indonesia, where the World Health Organization (WHO) classification is used, obese individuals were approximately 2.61 times more likely to have HTN than those with normal BMI, and those with abdominal obesity were 1.50 times more likely to have HTN than those without abdominal obesity, after adjusting for covariates [7]. Our findings are consistent with a study conducted in 13 African countries (H3Africa Cardiovascular Innovation Resource), which found that obesity is significantly associated with a more than twofold increase in HTN in men and women and plays a role across Africa [24].

Data from a study examining cardiovascular risk factors in seven cities from seven Latin American countries showed that the prevalence of HTN, diabetes and dyslipidemia was two to three times higher in obese people than in the normal weight population [34]. One study found that the odds of developing HTN increased 1.04 times with each additional centimeter (cm) of WC [12]. A cross-sectional study of more than 1,200 people in Goiania, Brazil, found that WC was significantly associated with HTN in both sexes [11]. The study showed that the prevalence of diabetes, HTN and dyslipidemia increased significantly with increasing BMI category [13]. Epidemiological data generally indicate that obesity or overweight is prevalent in hypertensive populations and that obesity can lead to HTN and cardiovascular disease [35]. Clinical studies show that maintaining a BMI of less than 25 kg/m^2^ is effective for primary prevention of HTN and weight loss lowers BP in most people with hypertension [8]. Assessments of general and central obesity are important to assess potential health risks.

The results of this study show that people with dyslipidemia have a higher risk of developing HTN. Several previous studies have investigated the association between dyslipidemia and HTN in non-Asian populations and have shown that dyslipidemia is closely associated with the development of HTN [36, 37]. In addition, a study in adult Chinese men showed that higher TC, LDL-C and non-HDL levels were strongly associated with the incidence of HTN [6]. A study by Otsuka et al. based on data from 14,215 male workers without hypertension who were followed up for 4 years to detect new-onset HTN showed that elevated TC, LDL-C, and non-HDL-C levels were associated with an increased risk of hypertension [38]. The results of He et al. showed that higher TG and lower HDL-C were associated with a higher risk of new-onset HTN, but for TC, LDL-C, and non-HDL-C, the risk of new-onset HTN was increased only at normal levels in a Chinese community-based cohort [39]. In a Taiwanese population, high TC/HDL-C, low HDL-C, and high TG were associated with a higher risk of HTN [40]. Furthermore, in a population-based cohort of middle-aged men who did not have HTN at baseline, a 7-year follow-up reported that a 1 SD increase in TG levels was associated with a 1.6-fold [95% CI: 1.2–2.3] increased risk of developing HTN, whereas HDL-C levels had a protective effect [23]. Cohort studies have shown that dyslipidemia leads to HTN in apparently healthy individuals. Therefore, HTN may be the result of dyslipidemia or closely associated metabolic abnormalities [23, 39].

Few studies have investigated whether there is an interaction between dyslipidemia and obesity in HTN. A study in China with a population of 2740 individuals confirmed the interaction effects of BMI and dyslipidemia on the risk of HTN [41]. In addition, another study in China showed that triglyceride glucose (TyG) index and obesity had a significant increasing interaction effect on blood pressure [42]. Therefore, based on the results of this study that obesity and dyslipidemia have a synergistic effect with HTN and are important risk factors for cardiovascular disease, further studies on the interaction mechanism between these factors are needed.

This study has several strengths, including that it is the first community-based cross-sectional study with a large sample size (40,387 participants) that examined the synergistic effect of obesity and dyslipidemia on HTN. In addition, the data of this study were collected with standardized questionnaires by a trained and certified team, which increases the reliability of the data. However, there were also limitations. First, we lacked information on medications that may affect HTN and dyslipidemia, which may have led to an underestimation of the association between hypertension and lipid profile. Information on demographic data, lifestyle and risk factors such as smoking was obtained by interviewing respondents, which may lead to information bias. In addition, the data analysis was based on cross-sectional studies, which cannot predict causality.

## Conclusion

Obesity and dyslipidemia are risk factors for HTN. In addition, dyslipidemia with central obesity increases the risk of HTN and has a synergistic interaction effect on HTN. Therefore, the coexistence of obesity and lipid abnormalities has many clinical implications and should be appropriately monitored and evaluated in the management of HTN.

## Data Availability

Data is available by contacting the corresponding author.
